# Apoptosis Induction of Armeniacae Semen Extractin Human Acute Leukemia (NALM-6 and KG-1) Cells

**Published:** 2019-07-01

**Authors:** Shiva Mosadegh Manshadi, Maliheh Safavi, Shahrbano Rostami, Fatemeh Nadali, Mohammad Reza Shams Ardekani

**Affiliations:** 1 Department of Hematology and Blood Banking, School of Allied Medical Sciences, Tehran University of Medical Sciences, Tehran, Iran; 2 Department of Biotechnology, Iranian Research Organization for Science and Technology (IROST), Tehran, Iran; 3 Hematology-Oncology and Stem Cell Transplantation Research Center, Tehran University of Medical Sciences, Tehran, Iran; 4 Department of Pharmacognosy and Medicinal Plants Research Center, School of Pharmacy, Tehran University of Medical Sciences, Tehran, Iran

**Keywords:** Armeniacae semen, Acute leukemia, Acute leukemia cell lines, Caspase-3

## Abstract

**Background: **Prunusarmeniaca is a member of the Rosacea family. The most important ingredient of this family is amygdalin that is believed to have anti-tumor and analgesic properties. The aim of this study was to evaluate the anti-proliferative effects of Armeniacae semen extract on the acute leukemia, NALM-6, and KG-1 cell lines, and investigate the effect of the extract on apoptosis of these cell lines and caspase-3 gene expression.

**Materials and Methods: **We prepared aqueous, ethyl acetate, and hydro alcoholic extracts of the Armeniacae semen. The NALM-6 and KG-1 cell lines and mononuclear cells (PBMCs) of healthy controls were treated with different doses of the extracts for 48 hours, and then cell viability was investigated with the MTT test. High-Performance Liquid Chromatography was done for amygdalin identification. The percentage of apoptotic cells was determined using the Annexin V-FITC/PI flow cytometric kit, and caspase-3 gene expression was evaluated.

**Results: **MTT test revealed that the strongest Inhibition Concentration (IC50) in KG-1 and NALM-6 cell lines was related to the ethyl acetate extract. This extract did not have toxic effects on PBMCs. Flow cytometric analysis showed that the ethyl acetate extract at its IC50 concentration led to almost 50% apoptosis in both cell lines after 48 hours. In the molecular examination, after treatment, a significant increase was seen in caspase-3 gene expression in NALM6 and KG1 cells compared to the control (P<0.001 and P <0.05, respectively).

**Conclusion: **Our data confirmed that the ethyl acetate extract of Prunusarmeniaca could reduce the proliferation of KG-1 and NALM-6 cell lines probably by activating the apoptotic pathway.

## Introduction

 Acute leukemia refers to rapid and clonal proliferation of lymphoid and myeloid progenitor cells in the bone marrow  ^1^^, ^^2^ . Based on the type 

of stem cell involved, it is divided into two major groups: AML (acute myeloid leukemia) and ALL (acute lymphoid leukemia)^   3 ^. AML is the most common cause of acute leukemia in the first few months of life, in middle-aged people, and in the elderly, and has a prevalence of 10,000,000 per year in people over 60 years old    4 . ALL is the most common malignancy in childhood. ALL mostly occurs between the ages of 3 and 7 years^        5 ^. There is a secondary increase in the incidence of ALL in patients older than 40 years    6 . Its specific treatment is chemotherapy. Dissatisfaction with conventional treatments and side effects of chemotherapy are the most important reasons for use of natural drugs^7^^,^^8^. 

Rosaceous plants, which are widely distributed, produce different economically important products, including many edible fruits, as bitter almonds, apricots, peaches, plums, etc^   9 ^. The family has an important glycoside called amygdalin. This component decomposes under glycosidase reactions, releasing hydrocyanic acid and benzaldehyde. Hydrochloric acid is an anti-tumor compound and benzaldehyde has analgesic properties ^   10 ^. 

Amygdalin has an antitumor effect by settling carcinogens in the body, inhibiting the nutritional source of cancer cells, and blocking the growth of the tumor cells. It can also improve the symptoms of patients in the last stages of cancer and increase their survival^   9 ^. Many studies have confirmed anti-tumor properties of amygdalin. Hyun-Kyung Chang et al. (2005) showed that amygdalin induces apoptosis in bladder cancer cells^   7 ^. In 2005, Hae-Jeong Park et al. demonstrated that Armeniacae semen down-regulated special genes involved in the cell cycle in the colon cancer cell line^   11 ^. Hee-Young Kwon et al. (2003) showed that Persicae semen extract induces apoptosis in human promyelocytic leukemia (HL-60) cells^12^. Jasmina Makarevic et al. (2014) reported that amygdalin from apricot kernels affects bladder cancer cell adhesion and invasion in vitro^   13 ^. Because of these features and the lack of coherent studies on various types of leukemia, we decided to use the Armeniacae semen, a member of the Rosacea family, which contains large amount of the amygdalin, to evaluate its anti-proliferative effect on the acute leukemia, NALM-6 (ALL) and KG-1 (AML) cell lines. In addition, we investigated the effect of the Armeniacae semen on apoptosis of these cell lines and caspase-3 gene expression.

## MATERIALS AND METHODS

**Cell culture**

NALM-6 and KG-1 acute leukemia cell lines (ALL and AML, respectively), which were provided by the Pastor Institute of Iran, were grown and sub cultured in RPMI1640 containing 20mM HEPES-buffer and glutamax 1% (Biosera, France) supplemented with 10% heat-inactivated FBS (fetal bovine serum) (Gibco, USA) and 100µg/ml penicillin/streptomycin (Biosera). Mononuclear cells were isolated from the peripheral blood of healthy individuals using Ficoll-Paque. The cultures were incubated at 37^◦^C with 5% CO_2_ and 95% humidity. The medium was changed every 2-3 days.

**Extracts preparation**

Two hundred grams of the Armeniacae semen was hatched from the shell and dried in the shade for a week. The seeds were then crushed by a pounder. At first, the dry powder was macerated in a petroleum ether-solvent to remove oils to not disturb the cytotoxic test. After oil extraction, the seed powder was allowed to dry completely and the solvent evaporate. Then, 50 g of the powder was weighed to prepare an aqueous extract. For this purpose, the seed powder was macerated in 90 ° C water for 30-45 minutes. Then, 500 ml of ethyl acetate, was added to the rest of the powder. After 48 hours, the solvent was removed and a new ethyl acetate solvent was added again. The procedure was repeated 3 times. The above processes were also repeated for 60% methanol. The extracts from each step were filtered with filter papers, transferred to a rotary balloon (Heidolph, Germany) and concentrated at 100 rpm at 40 ° C.

**MTT assay**

To check cell viability, we used the 3-(4, 5-dimethylthiazol-2-yl)-2, 5 di-phenyl tetrazolium bromide (MTT) assay. The cells were seeded in a 96-well plate at a concentration of 5×10^4 ^cells/well. Both cell lines were treated with aqueous, methanol, and ethyl acetate extracts of the Armeniacae semen at concentrations of 0.125, 0.25, 0.5, 1 mg/ml for 48h. PBMCs were treated with the extract that had the best IC50 on the MTT test at concentrations of 0.125, 0.25, 0.5, 1 mg/ml for 48h. After this time, 50µl MTT (Sigma, USA) was added to each well and incubated for 4h. Then, the supernatant was removed and 100μl DMSO (Sigma-Aldrich, USA, Biologic Grade) was added to dissolve MTT. Following incubation for 15 minutes, the OD was read with an ELISA plate reader (BioTek ELx808, USA) at 492 nm. The assay was performed at least three times.

**SPE method**

A solid-phase extraction cartridge (Chromafix-Germany) was used to separate and concentrate the target material of the extract, including a mixture of different materials. At first, the cartridge was washed with 5 ml of methanol and activated with 5ml of water. Then, 5 ml of the extract at a concentration of 20 mg/ml was passed through the cartridge. After washing with 10% methanol aqueous solution, to remove co-adsorbents, the remaining analytes were collected on SPE with 5 ml of pure methanol.

**Amygdalin identification by reverse phase HPLC**

In order to Amygdalin identification in the extract that had the best IC50 on the MTT test, the gradient method was used to elute other components quickly and reduce the analytical time after amygdalin peak observation. For this purpose, the mobile phase was adjusted to 15: 85 methanol-water for 30 minutes and pure methanol after 30 minutes. The mobile phase was filtered before use with a vacuum filter system containing a 0.45μm filter (Millipore-Germany). Separation was conducted on a C_18 _column (250×4.6mm). The column temperature was 30°C, and the detection wavelength was set at 215 nm. At first, the amygdalin standard was injected to the Agilent 1260 chromatographic system, including a UV detector and ChemStation data system at a concentration of 100μg / ml and a volume of 20 μl, and then the exact location of the peak was determined. This action was repeated 3 times. Then, 20 μl of the specimen (isolated by SPE) was injected and after peak observation, the test was repeated 3 times. By comparing the peak area of the standard and sample, the percentage of amygdalin in the extract was obtained.

**Flow cytometry**

In order to determine the amount of primary and secondary apoptosis induced under the influence of the extract in each cell line, the cells were grown at a concentration of 1×10^6^ cell/ well on a 6-well plate. Then, the concentration of (the best) IC50 was added to each well. After 48 hours, flow cytometry was performed by the BD FACS Calibur Flow Cytometry Machine (BD Biosciences_ USA), and the results were analyzed using the FlowJo.7.6.1 software. We conducted flow cytometry measurements based on the protocols of eBioscience.

**RT-PCR**

RT-PCR was performed to identify caspase-3 gene expressions. For this purpose, the cells were collected after 48 hours of treatment with the extract that had the best IC50 on the MTT test, and their total RNA was extracted using the Trizol (Qiagen, Germany) method. Then, 1µg total RNA was converted to cDNA using the PrimeScript 1st strand cDNA Synthesis kit (Takara, Japan) according to kit manufacturer’s instructions. RQ-PCR assay was performed to investigate the level of caspase-3 gene expression in drug-treated and control groups using primers in Table 1. β-2 microglobulin (B2M) was used  as a housekeeping gene for normalization of RT-qPCR data .It should be noted that all tests were performed in triplicate. The fold change Casp3 mRNA in treated cells in comparison with untreated cells was computed by the 2-^ΔΔCT^ method.

**Table 1:** Primers used for qRT-PCR analysis

**Gene**	**Primer(**5′-3′)	**PCR product size ** **(bp)**
Casp3 (Forward) Casp3 (Reverse)	TCTGGTTTTCGGTGGGTGTG CGCTTCCATGTATGATCTTTGGTTC	137
B2M (Forward) B2M (Reverse)	CTCCGTGGCCTTAGCTGTG TTTGGAGTACGCTGGATAGCCT	69

**Statistical analysis**

All Experiments were performed in duplicate and repeated three times. IC50 was calculated using the Excel 2013 software, and flow cytometry analysis was done using the FlowJo.7.6.1 flow cytometry software. The data are expressed as mean ± SD for all experiments. GraphPad Prism 5.0 (GraphPad Software, Inc., San Diego, CA) was used to detect significant differences between the control and treated groups. Statistical significance was defined at *P<0.05, **P<0.01, and ***P<0.001 compared to the corresponding controls.

## Results

**Ethyl acetate extract had the best IC50 on the MTT test**

**Figure 1:**
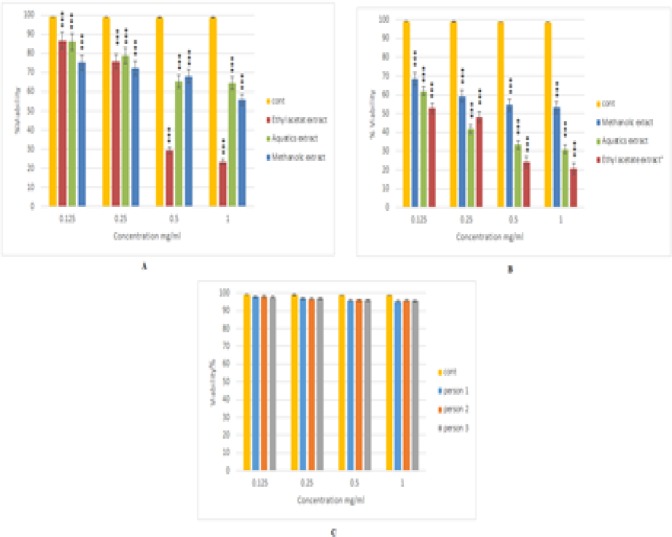


When cells were treated with the aqueous methanol, and ethyl acetate extracts of the Armeniacae semen at concentrations of 0.125, 0.25, 0.5, 1 mg/ml for 48h, the MTT test showed that cell proliferation was inhibited in a dose-dependent manner with all extracts (Figure1). The best IC50 was related to ethyl acetate extract of the Armeniacae semen in both Kg1 (0.159 mg/ml) and Nalm6 (0.388 mg/ml) cell lines. This extract at the concentrations of 0.125, 0.25, 0.5, 1 mg/ml for 48h did not show significant cytotoxic effects on PBMCs compared to the untreated control (Figure1).

**Amygdalin identification by HPLC**

The results of the HPLC test indicated the presence of amygdalin in the ethyl acetate extract of Armeniacae semen. There was 0.67% amygdalin in this extract that was calculated as follows: Area sample/ Area STD×C STD.

**Figure 2:**
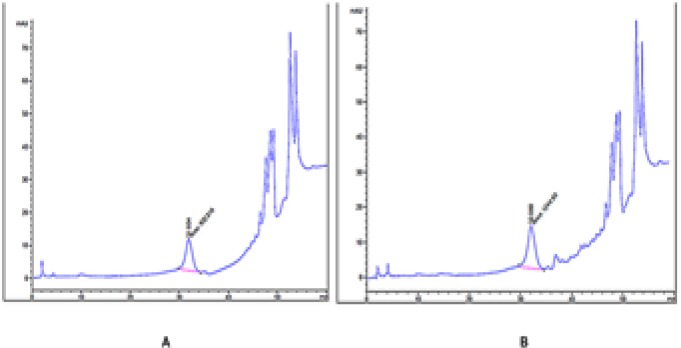


**Analysis of apoptosis by double staining KG-1 and NALM-6 cells with annexin V-FITC and PI**

KG-1 and NALM-6 cells became apoptotic after they were treated with the ethyl acetate extract of Armeniacae semen. The distribution of apoptotic cells measured by flow cytometry showed that the percentage of early (Q3) and late-apoptotic cells (Q2) was 63±1.48% and 32.7±2.31% in KG-1 and 59.22±1.43% and 30.61±1.94% in NALM-6 cell lines, respectively.

**Figure 3:**
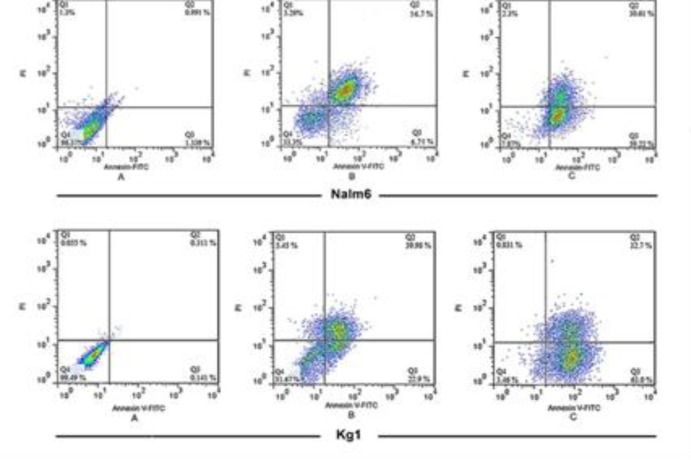


**Effect of Ethyl acetate extract of Armeniacae semen on casp3 gene expression**

Molecular examination 48 hours after treatment of KG-1 and NALM-6 cell lines showed a significant increase in caspase-3 gene expression in test samples compared to the controls. (*p*<0.05 in KG1 and *p*<0.001 Nalm6 compared to the untreated control cells.)

**Figure 4:**
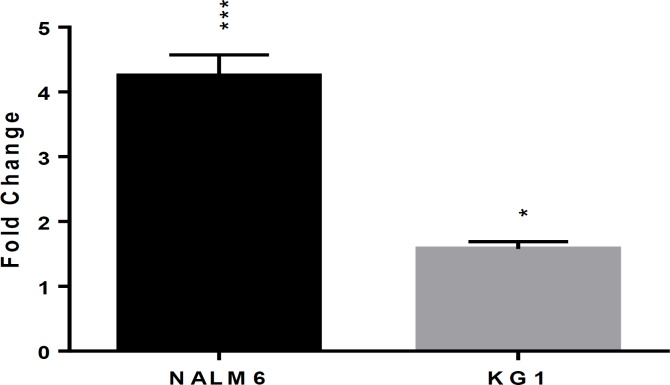


## Discussion

 The main purpose of this study was to evaluate whether Prunus armeniaca extract causes apoptosis in NALM-6 (acute lymphatic leukemia) and KG-1 (acute myeloid leukemia) cell lines. 

In the present study, based on the evaluation of cell survival by the MTT assay, the cell viability in both KG-1 and NALM-6 cell lines was dose dependent and the survival rate of the cells reduced, with an increase in the concentration of the mixture. Moreover, the best IC50 was related to the ethyl acetate extract in both cell lines. The effect of the extract on the cell viability seems to be dependent on the cell type. Our results are in line with other studies, showing cytotoxic effect of amygdalin on various cancer cell types ^10^^,^^11^^,^^14^^,^^15^ . In 2005, Park HJ et al. reported that the extract from the amygdalin has a dose-dependent cytotoxic effect on human colon cancer (SNU-C4)^   11 ^. They also reported that treatment of human chronic myeloid leukemia (K562 cell line) with amygdalin at different concentrations resulted in decrease in cell viability in a dose-dependent manner ^   14 ^. In other studies, cytotoxic effect of amygdalin was shown on prostate cell lines (DU145 and LNCaP) and human cervical cancer HeLa cells ^10^^,^^15^.

Since most anticancer and cytotoxic compounds induce apoptosis in tumor cells, a quantitative study of apoptosis was performed in both KG-1 and NALM-6 cell lines treated with the ethyl acetate extract of Armeniacae semen. Analysis of flow cytometric results in both cell lines indicated an almost 50% apoptosis at IC50. Moreover, investigation of caspase-3 (executive pathway) gene expression at IC50 showed a significant increase in both cell lines compared to controls. The other studies involving other cancerous cell lines also confirmed apoptosis induction and increased caspase-3 activity by amygdalin ^7^^,^^10^^,^^15^^,^^16^ . Evaluation of the treatment of UMUC-3, TCCSUP, and RT-112 bladder cancer cell lines with amygdalin at concentrations of 1, 10, 25 mg/ml using the Annexin-V-FITC/PI kit showed  a dose-dependent relationship between amygdalin concentration and Annexin V positive cells^   7 ^ . 

The key role of Caspase-3 in apoptotic pathways is well established. Therefore, the mRNA expression level of Casp3 was assessed to evaluate the effect of the amygdalin extract on the induction of cell death. Our gene expression study showed that the ethyl acetate extract of Armeniacae semen increases the Casp3 at mRNA level. Chang et al. showed that amygdalin increased caspase-3 activity significantly in both DU145 and LNCaP prostate cancer cell lines^   10 ^. Lee et al. showed that amygdalin induces apoptosis in Hs578T breast cancer cells through the caspase-3 pathway ^   16 ^. 

The effect of the ethyl acetate extract of Armeniacae semen on PBMCs isolated from normal blood at different concentrations did not show significant cytotoxic effects compared to the negative control. There are hypotheses about the mechanisms of the differences in the response of normal and cancerous cells to amygdalin. Some studies suggest that the cancer cells are rich in β-glucosidase, which is capable of digesting amygdalin to produce cyanide, leading to toxic effects on cancer cells^17^^,^^18^. Some other studies suggest that rhodanase, which is capable of eliminating cyanide toxicity, exist in normal tissues, but is ineffective in cancer cells. Combined activity of the two enzymes may be responsible for inducing toxic effects in amygdalin-treated cancer cells, while normal cells remain unaffected^11^^,^^19^.

## CONCLUSION

 Taken together, the results of the present in vitro study (which never was done before on these types of leukemia) suggest that the ethyl acetate extract of Armeniacae semen have anticancer effects on AML and ALL. However, future studies should focus on the isolation of the effective ingredients of the ethyl acetate extract and evaluation of their effects on the KG-1 and NALM-6 cell lines in human and animal models as well as the assessment of the effect of the extract on the cell cycle. In addition to measuring the expression of caspase-3 gene, the activity of this enzyme should be assessed through the caspase-3 activity measurement kit.

## References

[1] Elaine M Keohane, Larry J Smith, Jeanine M Walenga ( 2016). Rodak’s Hematology Clinical Principles and Applications.

[2] Mohammadi S, Nikbakht M, Sajjadi SM (2017). Reciprocal Interactions of Leukemic Cells with Bone Marrow Stromal Cells Promote Enrichment of Leukemic Stem Cell Compartments in Response to Curcumin and Daunorubicin. Asian Pac J Cancer Prev.

[3] William B Ershler, Kenneth Kaushansky, Marshal A Litchtman, Josef T Prchal, Marcel M Levi, Oliver W Press, Linda J Burns, Michael A Caligiuri (2016). Williams Hematology.

[4] Richard A McPherson, Matthew R Pincus (2017). Henry's Clinical Diagnosis and Management by Laboratory Method.

[5] Nikbakht M, Jha AK, Malekzadeh K (2017). Aberrant promoter hypermethylation of selected apoptotic genes in childhood acute lymphoblastic leukemia among North Indian population. Exp Oncol.

[6] A Victor Hoffbrand, Paul AH Moss ( 2016). Hoffbrand’s Essential Haematology.

[7] Makarević J, Rutz J, Juengel E (2014). Amygdalin blocks bladder cancer cell growth in vitro by diminishing cyclin A and cdk2. PloS one.

[8] Mohammadi S, Ghaffari SH, Shaiegan M (2016 J). Curcumin Veto the Effects of Osteopontin (OPN) Specific Inhibitor on Leukemic Stem Cell Colony Forming Potential via Promotion of OPN Overexpression. Int J Hematol Oncol Stem Cell Res.

[9] Song Z, Xu X (2014). Advanced research on anti-tumor effects of amygdalin. J Cancer Res Ther.

[10] Chang H-K, Shin M-S, Yang H-Y (2006). Amygdalin induces apoptosis through regulation of Bax and Bcl-2 expressions in human DU145 and LNCaP prostate cancer cells. Biol Pharm Bull.

[11] Park HJ, Yoon SH, Han LS (2005). Amygdalin inhibits genes related to cell cycle in SNU-C4 human colon cancer cells. World J Gastroenterol.

[12] Kwon HY, Hong SP, Hahn DH (2003). Apoptosis induction of Persicae Semen extract in human promyelocytic leukemia (HL-60) cells. Arch Pharm Res.

[13] Makarević J, Rutz J, Juengel E (2014). Amygdalin influences bladder cancer cell adhesion and invasion in vitro. PloS One.

[14] Park HJ, Lee SK, Baik HW (2006). Amygdalin modulates cell cycle regulator genes in human chronic myeloid leukemia cells. Mol Cell Toxicol.

[15] Chen Y, Ma J, Wang F (2013). Amygdalin induces apoptosis in human cervical cancer cell line HeLa cells. Immunopharmacol Immunotoxicol.

[16] Lee HM, Moon A (2016). Amygdalin Regulates Apoptosis and Adhesion in Hs578T Triple-Negative Breast Cancer Cells. Biomol Ther (Seoul).

[17] Dorr RT, Paxinos J (1978). The current status of laetrile. Ann Intern Med.

[18] McCarty MF (1980). The nutritionally and metabolically destructive" nutritional and metabolic antineoplastic diet" of laetrile proponents. Am J Clin Nutr.

[19] Newmark J, Brady RO, Grimley PM (1981). Amygdalin (Laetrile) and prunasin beta-glucosidases: distribution in germ-free rat and in human tumor tissue. Proc Natl Acad Sci U S A.

